# Endometrial polyps are non-neoplastic but harbor epithelial mutations in endometrial cancer drivers at low allelic frequencies

**DOI:** 10.1038/s41379-022-01124-5

**Published:** 2022-07-07

**Authors:** Subhransu S. Sahoo, Mitzi Aguilar, Yan Xu, Elena Lucas, Valerie Miller, Hao Chen, Wenxin Zheng, Ileana C. Cuevas, Hao-Dong Li, David Hitrys, Megan B. Wachsmann, Justin A. Bishop, Brandi Cantarell, Jeffrey Gagan, Prasad Koduru, Jeffrey A. SoRelle, Diego H. Castrillon

**Affiliations:** 1 Department of Pathology, UT Southwestern Medical Center, Dallas, TX USA; 2 Next Generation Sequencing Clinical Lab, UT Southwestern Medical Center, Dallas, TX USA; 3 Harold C. Simmons Comprehensive Cancer Center, UT Southwestern Medical Center, Dallas, TX USA; 4 Department of Obstetrics and Gynecology, UT Southwestern Medical Center, Dallas, TX USA; 5 Molecular Machines & Industries, Haslett, MI USA; 6 Veterans North Texas Health Care System, Dallas, TX USA; 7 Lyda Hill Department of Bioinformatics, UT Southwestern Medical Center, Dallas, TX USA

**Keywords:** Translational research, Endometrial cancer

## Abstract

Endometrial polyps (EMPs) are common exophytic masses associated with abnormal uterine bleeding and infertility. Unlike normal endometrium, which is cyclically shed, EMPs persist over ovulatory cycles and after the menopause. Despite their usual classification as benign entities, EMPs are paradoxically associated with endometrial carcinomas of diverse histologic subtypes, which frequently arise within EMPs. The etiology and potential origins of EMPs as clonally-derived neoplasms are uncertain, but previous investigations suggested that EMPs are neoplasms of stromal origin driven by recurring chromosomal rearrangements. To better define benign EMPs at the molecular genetic level, we analyzed individual EMPs from 31 women who underwent hysterectomy for benign indications. The 31 EMPs were subjected to comprehensive genomic profiling by exome sequencing of a large panel of tumor-related genes including oncogenes, tumor suppressors, and chromosomal translocation partners. There were no recurring chromosomal rearrangements, and copy-number analyses did not reveal evidence of significant chromosome-level events. Surprisingly, there was a high incidence of single nucleotide variants corresponding to classic oncogenic drivers (i.e., definitive cancer drivers). The spectrum of known oncogenic driver events matched that of endometrial cancers more closely than any other common cancer. Further analyses including laser-capture microdissection showed that these mutations were present in the epithelial compartment at low allelic frequencies. These results establish a link between EMPs and the acquisition of endometrial cancer driver mutations. Based on these findings, we propose a model where the association between EMPs and endometrial cancer is explained by the age-related accumulation of endometrial cancer drivers in a protected environment that—unlike normal endometrium—is not subject to cyclical shedding.

## Introduction

Endometrial polyps (EMPs) are localized, well-circumscribed growths that protrude into the uterine cavity. They are composed of benign-appearing epithelial (glandular) and stromal (fibroblastic) elements admixed with blood vessels, with variable ratios of the epithelial vs. stromal components. EMPs are common pathologic findings and may be isolated or occur as multiple discrete lesions^1^. EMPs have high prevalence in pre- and postmenopausal women, with an overall prevalence of 8–35%^2,3^. Abnormal uterine bleeding is the most frequent presenting symptom^3^. The prevalence of EMPs is increased in infertile women, suggesting that EMPs contribute to infertility, and hysteroscopic removal improves fertility outcomes^4^.

Despite their ubiquity, the etiology of EMPs is unknown. Risk factors include age, obesity, and tamoxifen use^1,3,5,6^. Paradoxically, EMPs are considered benign, but yet are associated with cancer. Endometrioid adenocarcinoma and its precursor atypical hyperplasia/endometrioid intraepithelial neoplasia (AH/EIN) is a common incidental finding in EMPs. Case series have reported a wide range in the risk of malignancy in EMPs (0–15%) with one meta-analysis reporting a prevalence of malignancy in EMP of 2.7%^7^. Endometrial serous carcinoma, which has distinct epidemiologic risk factors^8^ is often confined to an EMP, indicating that the serous carcinoma arose in a pre-existing polyp^9^. In one series, 31% of stage 1 endometrial serous cancers were confined to an EMP^10^. Other histologic endometrial cancer subtypes, including clear cell adenocarcinoma and carcinosarcoma, can also be confined to EMPs^11–15^. Because of their associations with abnormal uterine bleeding, infertility, and cancer, many clinicians believe that all EMPs should be hysteroscopically resected^1,16^.

As discrete tumors, EMPs have features of neoplasms driven by somatically-acquired genetic alterations. The poorly understood link between EMP and endometrial cancer also raises questions about the status of EMPs as potential neoplasms driven by unknown genetic alterations. Studies in the 1990s reported chromosomal rearrangements involving 6p21, such as t(6;20)(p21;q13) and t(6;14)(p21;q24)^17–20^, among other reports of chromosomal rearrangements in EMPs^21–23^. Several of the studies concluded that the rearrangements occurred in the mesenchymal component. However, one limitation of classical cytogenetics through karyotyping is the occurrence of spontaneous chromosomal rearrangements that are tissue culture artifacts. Although independent validation of chromosomal rearrangements through other means (e.g., break-apart FISH) is now straightforward^24^, this method was not available at the time of these early reports, and there have not been further published explorations of a potential genetic basis of EMPs in the intervening years.

In this study, we took advantage of recent advances to revisit the molecular genetic origins of EMPs and their status as potential clonally-derived neoplasms. Thirty-one EMPs were subjected to systematic genetic analysis through a comprehensive cancer gene panel in clinical use. Given the presence of abundant mesenchymal and epithelial components in EMPs and their variable ratios, either could be the cellular component driving growth if EMPs were indeed clonally-derived neoplastic outgrowths, with the other component being “recruited”. Our approach was designed to detect alterations in DNA (for detection of somatic point mutations) and RNA (for identification of chromosomal rearrangements including translocations and gene fusions that typify mesenchymal neoplasms) with high sensitivity and specificity.

## Materials and methods

### Case selection

After approval from the UT Southwestern IRB, we retrospectively identified cases of patients ≥50 y/o via text searches for a final diagnosis of (benign) EMP accessioned between 2010 and 2020 at the UT Southwestern Clements University Hospital. Standard histologic criteria were used for the initial diagnosis of EMP including the presence of glandular and mesenchymal elements and exclusion of other lesions (e.g., submucous leiomyomata) that can grossly resemble EMP. A clinical or histopathological diagnosis of EMP prior to the hysterectomy was not a selection criterion. Cases with concurrent or prior diagnosis of AH/EIN in the EMP or endometrium or any uterine malignancy were excluded. A prior diagnosis of cancer in an unrelated organ system was not an exclusion criterion. All cases were reviewed to confirm the presence of an EMP and absence of uterine malignancy/premalignancy. All patients had a single EMP. Criteria for AH/EIN in an EMP included cribriforming and cytologic distinctiveness. EMPs with gland:stromal ratios >1 were permitted since EMPs can exhibit greater gland crowding than normal endometria^25,26^. However, none of the cases raised concerns for AH/EIN either in the original diagnoses or upon secondary review.

### UTSW clinical NGS panel

Unstained 4 μm sections were cut from one tissue block for each case. Areas of non-EMP (i.e., subjacent endomyometrium) were macrodissected away with a blade. DNA was also prepared from control somatic (i.e., “germline”) tissues removed during the hysterectomy, such as ovary or cervix.

DNA and RNA were isolated using Qiagen Allprep kits. The custom panel of DNA probes was used to produce an enriched library, using Kapa Biosystems and Illumina chemistry, containing all exons for the 1516 cancer-related genes (Table S1), which were sequenced on an Illumina NextSeq 550 instrument. DNA and RNA sequence analyses were done using custom germline, somatic and mRNA bioinformatics pipelines run on the UTSW Bio-High Performance Computer cluster and optimized for detection of single nucleotide variants, indels and gene fusions^27,28^. Median target exon coverage for the assay is 900X with 94% of exons at 100x. FASTQ files were aligned into BAM files with BWA, then called with a combination of variant callers for somatic variants (platypus, GATK, SAMtools), copy number variants (OncoCNV), and fusions (PINDEL, StarFusion) to create a VCF file, which was annotated through ANSWER software^28^. The RNA panel covers 1505 genes and can capture unbaited partners. The limit of detection is 5 RNA reads. Gene Set Cancer Analysis to compare the spectrum of mutations to TCGA data sets was performed on the GSCA portal http://bioinfo.life.hust.edu.cn/GSCA/#/mutation^29^ and cBioPortal and plotted with the Cancer Type Summary Tools^30^.

### Quantitative analysis of epithelial:stromal ratios

EMP tissue sections were immunohistochemically stained with PAX8 to specifically label epithelial cells. For PAX8 IHC, 4 μm tissue sections were dried for 1 h at 60 °C. Deparaffinization, rehydration, and target retrieval were performed per manufacturer’s instructions followed by incubation with prediluted monoclonal mouse PAX8 antibody *(*clone MRQ-50, Cell Marque) for 30 min. Slides were rinsed in wash buffer for 5 min, incubated with EnVision™ FLEX HRP reagent for 20 min at room temperature, and then in 3,3’-diaminobenzidine (DAB) tetrahydrochloride chromogen for 10 min at room temperature. Slides were counterstained for 5 min with hematoxylin. Mounting was performed using nonaqueous permanent mounting media.

The stained slides were scanned on an Aperio ScanScope CS. Representative areas were selected for quantification. The area of the epithelial cells was quantified by measuring the sum area of stained PAX8 cells in the selected region with ImageJ (National Institutes of Health, USA)^31^. The ImageJ threshold tool was used to adjust the signal from PAX8 stained cells to reduce background signal (representative images shown in Fig. 4A). % epithelium was calculated by dividing the total epithelial area by the area of the quantitated field (7.25 × 4.31 mm = 31.25 mm^2^).

### Use of immunohistochemical 3-marker AH/EIN panel

Immunohistochemistry for Pax2, Pten, and β-catenin and interpretation of marker aberrance was performed and cases scored as described^32^. The *n* = 79 normal controls and *n* = 111 AH/EIN were previously reported^32^.

### Laser capture microdissection and DNA isolation

Tissue sections (10 µm) were cut with a Leica Microtome and placed on polyethylene terephthalate membrane slides. Laser capture microdissection (LCM) was performed using the CellCut System with dissected cells collected onto the adhesive lid of 500 µl CapSure tubes (Molecular-Machines.com). Regions for LCM were selected from across the entire tissue sections, to obtain representative DNA from the epithelial vs. stromal compartments across each EMP section. DNA was prepared using the PicoPure DNA Extraction Kit (Applied Biosystems # KIT0103) per the manufacturer’s instructions.

### Digital PCR (dPCR)

For dPCR, 20 µl of the reaction mixture (dPCR supermix [Bio-Rad #1863023], mutant/wild-type probes and DNA) and 70 µl of droplet generation oil (Bio-Rad #1863005) were mixed by a droplet generator (Bio-Rad) to generate approximately 10,000 droplets. These droplets were amplified using dPCR mutation detection assays (mixture of dPCR mutant probe, FAM and wild-type probe, HEX) on a dPCR system through 35 amplification cycles; the PCR product was analyzed on a droplet reader using QuantaSoft software (Bio-Rad). Presence or absence of mutation in DNA samples was determined by signals from either mutant or wild-type probes. The signal output of mutant and wild-type alleles recorded as number of events was used to determine the mutant allele percentage in EMP DNA samples per the equation mutant allele (%) = FAM events/(FAM events + HEX events) X 100. The mutation detection assays (Bio-Rad) used were: *AKT1 p.E17K* (Bio-Rad #dHsaMDV2010031), *FBXW7 p.R465C* (#dHsaMDV2510506), *FBXW7 p.R465H* (#dHsaMDV2516800), *FGFR2 p.S252W* (#dHsaMDV2010045), *HRAS p.G12S* (#dHsaMDV2510568) and *KRAS p.G12S* (#dHsaMDV2510588).

### Fluorescence in situ hybridization (FISH)

FISH was performed on 4 µm tissue sections of a randomly-selected subset of *n* = 16 EMP cases using break-apart FISH probes (Empire Genomics) for *HMGA1* (#HMGA1BA-20-ORGR) and *HMGA2* (#HMGA2BA-20-ORGR). For both probes the 5ʹ fragment was labeled with TAMRA (orange signal) and the 3ʹ fragment labeled with fluorescein (green signal). FISH was performed per the manufacturer’s instructions.

## Results

### Genomic characterization of benign EMPs shows frequent mutations in endometrial cancer drivers

**Fig. 1:**
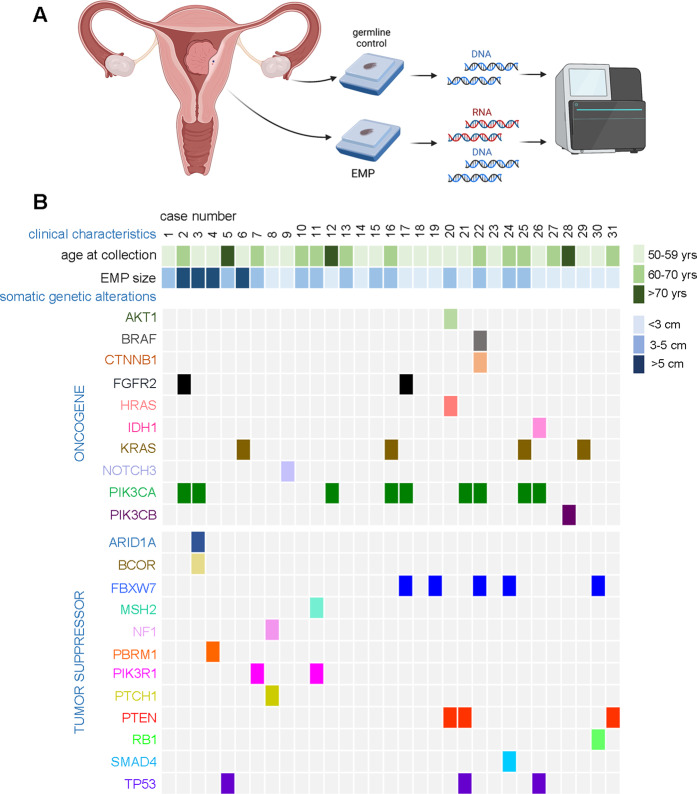
Study design and genomic analysis of benign EMPs. **A** Schematic of overall experimental strategy for genomic analysis of EMPs. **B** Case matrix of clinical parameters and mutations in EMPs from 31 patients.

We retrospectively identified *n* = 31 cases of hysterectomies performed for benign indications where a definitive EMP was grossly identified in the hysterectomy following the surgery. To exclude lesions of questionable etiology and biological significance, 1.5 cm was the minimum size cutoff. Clinical features of the selected cases are summarized in Table 1. Three of the patients had a history of tamoxifen administration for breast ductal carcinoma-in-situ or invasive ductal carcinoma (Table S2; see also discussion). Study of hysterectomies permitted (1) comprehensive gross evaluation of the entire endometrium permitting definitive identification of EMPs and (2) use of adjacent normal tissues for control DNA. Samples were subjected to a custom comprehensive NGS cancer panel (1516 genes) developed at the CLIA-certified UT Southwestern NGS Laboratory. This panel is used for the clinical management of cancer patients through the detection of gene fusions (RNA) and single nucleotide variants (DNA/RNA)^27^. The capture panel includes genes selected on the basis of their known or potential involvement in diverse chromosomal rearrangements resulting in gene fusions (Table S1), e.g., in sarcomas, leukemias, and lymphomas^33–35^. The workflow is schematized in Fig. 1A.

**Table 1:** Clinical features of selected cases.

EMP size:	1.5–9.5 cm (mean 3.1 cm)
Age range:	52–80 y/o (mean 60.8 y/o)
Reason for hysterectomy:	*N*
Thickened endometrium	2
Prolapse	9
Abnormal uterine bleeding	11
EMP	1
Leiomyomata	8
Total	31

Quality metrics were met for all DNA and RNA samples except for one EMP due to RNA degradation; this sample (EMP6) was subjected to DNA analysis only. Only one candidate gene fusion (*MYH9::SNHG16*, chromosomes 22:17) was detected among the remaining 30 cases (EMP7, Table S2). However, this putative fusion involved only <5% of the total reads, and the 3’ partner *SNHG16* encodes a long non-coding RNA. These observations make this rearrangement, which has not been reported in the literature and occurred only once in the 30 analyzable EMPs, of indeterminate biological significance. We did not identify any gene fusions involving chromosome 6p as previously reported^17–23^, or any other recurrent gene fusions likely to represent recurring EMP drivers. Break-apart FISH assays for *HMGA1* and *HMGA2* performed on a randomly-selected subset of *n* = 16 EMPs did not show any rearrangements in either the epithelial or stromal compartments (Fig. S1). Copy number analysis of the combined DNA on- and off-target reads in the 31 EMPs using CNVKit^36^ did not reveal clearly significant or recurring regions of amplification or deletion including *CDK4* and *MDM2* (Fig. S2).

All single nucleotide variants (SNVs) were tabulated and then classified per OncoKB^37^ as known oncogenic drivers versus alterations of uncertain functional significance (Table S2). Subsequent analyses were based on the known driver mutations, of which 46 were identified among the 31 EMPs (average 1.5 definitive oncogenic driver mutations/EMP). These somatically-acquired genetic alterations along with the clinical characteristics for each case are summarized in a case matrix (Fig. 1B). Mutations were identified in both oncogenic (gain-of-function) and tumor suppressor (loss-of-function) cancer drivers. Among the mutated genes there was a preponderance of well-established and common endometrial cancer drivers, such as *AKT1*, *ARID1A*, *BCOR*, *FBXW7*, *KRAS*, *PIK3CA*, *PIK3R1*, *PTEN*, *and TP53*, of note because the cancer gene panel is large/comprehensive and designed for systematic detection of cancer drivers across human malignancies, not just endometrial or gynecologic malignancies. Also, many of the mutations were canonical oncogenic drivers (e.g., *AKT1 p.Glu17Lys*, *FBXW7 p.Arg465His*, *KRAS p.Gly12Val*) (Table S2).

**Fig. 2:**
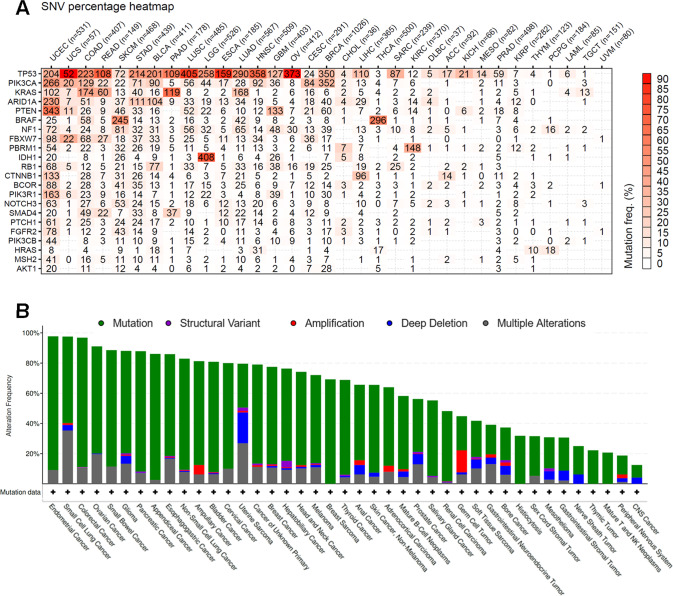
Assessment of similarity of mutational spectra in 31 EMPs relative to TCGA and MSK-Impact human cancer data sets. **A** Cancer gene set analysis showing that the mutation set most closely resembles TCGA-UCEC (uterine corpus endometrial carcinoma) among all TCGA data sets. UCS uterine carcinosarcoma, COAD colon adenocarcinoma; for remaining abbreviations see https://gdc.cancer.gov/resources-tcga-users/tcga-code-tables/tcga-study-abbreviations. **B** cBioPortal analysis of MSK-IMPACT data sets showing that among 41 different tumor types, endometrial cancer most closely matches the mutational spectrum identified in EMPs.

To formally assess these findings, the set of mutant alleles was related to all of the TCGA data sets for diverse human malignancies^29^. This unbiased analysis identified the Uterine Corpus Endometrial Carcinoma (TCGA-UCEC) as the human tumor type whose mutational spectrum most closely matched the identified mutations in EMPs, with the uterine carcinosarcoma (TCGA-UCS) dataset coming in second place (Fig. 2A). With a separate platform and dataset (MSK-IMPACT), endometrial cancer similarly emerged as the cancer type with the highest percentage of mutations in the identified genes (97.7%) (Fig. 2B)^38^. Thus, there was a non-random distribution of cancer driver mutations in EMPs. We conclude that mutations in canonical endometrial cancer drivers are common in EMPs, and that the overall spectrum resembles that of endometrial cancers. These findings establish a link between EMPs and the acquisition and persistence of endometrial cancer driver mutations.

### Low variant allelic frequencies indicate that endometrial cancer driver mutations are late events in EMPs

**Fig. 3:**
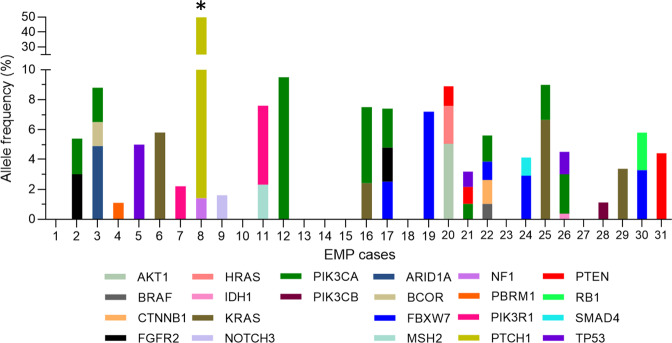
Variant allele frequencies for all mutations identified in 31 EMPs. One mutation (*PTCH1*) (asterisk) was found to be of germline origin and with concordantly higher VAF.

**Fig. 4:**
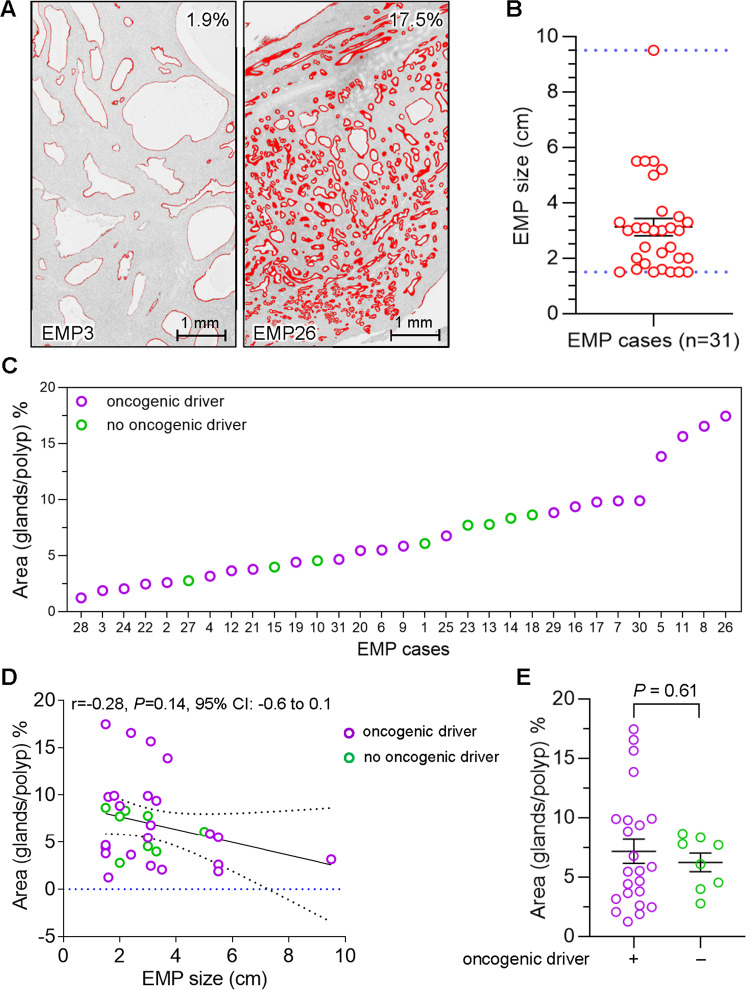
Characterization of morphological and histological features in EMP relative to presence of oncogenic drivers. **A** Representative ImageJ depictions of EMPs with differing epithelial:stromal ratios. The red lines demarcate the area of the epithelial compartment, i.e., glands, and do not include the luminal empty spaces (see methods for additional details). The percentage comprised by the epithelial cell compartment relative to the total area is shown in the upper right-hand corners. Scale bar = 1 mm. **B** Scatter dot plot of EMP size (*n* = 31). Bars represent mean ± SEM. **C** Presence/absence of oncogenic driver mutation in each EMP shown in order of ascending epithelial:stromal ratios (% epithelial area). **D** Scatter plot of EMP size correlation to % epithelial area. Pearson’s r determines correlation between data groups. **E** Scatter dot plot shows % epithelial area in EMPs (*n* = 31) with or without known oncogenic drivers. Bars represent mean ± SEM, *P* = 0.61, *t*-test.

Next, we analyzed the variant allele frequencies (VAFs) for each of the mutations. VAFs were 1–5% or lower, with only a small number of the somatic point mutations (8/46, 17.4%) in the 5–10% range (Fig. 3). Only 1 of 46 mutations (*PTCH1 p.PROfs*) exceeded a VAF of 10%, with a VAF of 48.4% (Fig. 3, asterisk). This mutation (in EMP8) was a 1 bp deletion resulting in a very early frameshift of the >1000 amino acid Patched protein, and thus represents a complete-loss-of-function allele. This allele was also detected in the control DNA sample with a germline detection filter for clinically actionable cancer predisposition genes per the American College of Medical Genetics and Genomics^39^ that is part of the UTSW Clinical NGS Panel workflow. The VAF close to 50% for this *PTCH1* allele is also consistent with its presence in the germline. Inherited *PTCH1* loss-of-function mutations result in Gorlin syndrome (a.k.a. basal cell nevus syndrome), an autosomal dominant familial cancer syndrome characterized by basal cell carcinomas among other abnormalities^40^. Although Gorlin syndrome has one well-established manifestation in the female reproductive tract (ovarian fibromas sometimes associated with prominent ascites), EMPs have not been reported in Gorlin syndrome. EMP8 had the second highest gland:area ratio (Fig. 4C) and was of unremarkable size (2.4 cm). Thus, the connection between the germline *PTCH1* mutation and EMP genesis in this patient is unclear, and may be incidental given the high incidence of EMPs in the general population^2,3^. In any case, the result establishes the NGS workflow’s ability to distinguish between variants of low versus high allelic frequencies and distinguish somatically-acquired vs. germline mutations. In summary, somatically-acquired mutations in EMPs are common but occur with consistently low VAFs, arguing (in concert with other data below) that they are not EMP-instigating clonal events but are acquired later in the developmental history of EMPs.

### Analysis of epithelial:stromal ratios across EMPs

Next, we studied variation in epithelial:stromal ratios among the EMPs. The percent area of the glandular (epithelial) component in each EMP was quantified by ImageJ based on Aperio ImageScope scans, showing that the percent epithelial component varied significantly among EMPs, as expected (Fig. 4A). EMP size ranged from 1.5 to 9.5 cm (average 3.1 cm) (Fig. 4B, Table S2). Plotting the EMPs in order of ascending epithelial:stromal ratios together with the presence or absence of oncogenic driver mutations failed to reveal a clear-cut relationship (Fig. 4C). There was also not a significant correlation between percent epithelial area and EMP size (*r* = −0.28, *P* = 0.14, CI: −0.6 to 0.1, Fig. 4D). Concordantly, there was not a statistically significant difference in epithelial component percentages in EMPs with or without known oncogenic drivers (*P* = 0.61, Fig. 4E).

### Analysis of EMPs with established 3-marker AH/EIN panel shows increased prevalence of Pax2 loss

**Fig. 5:**
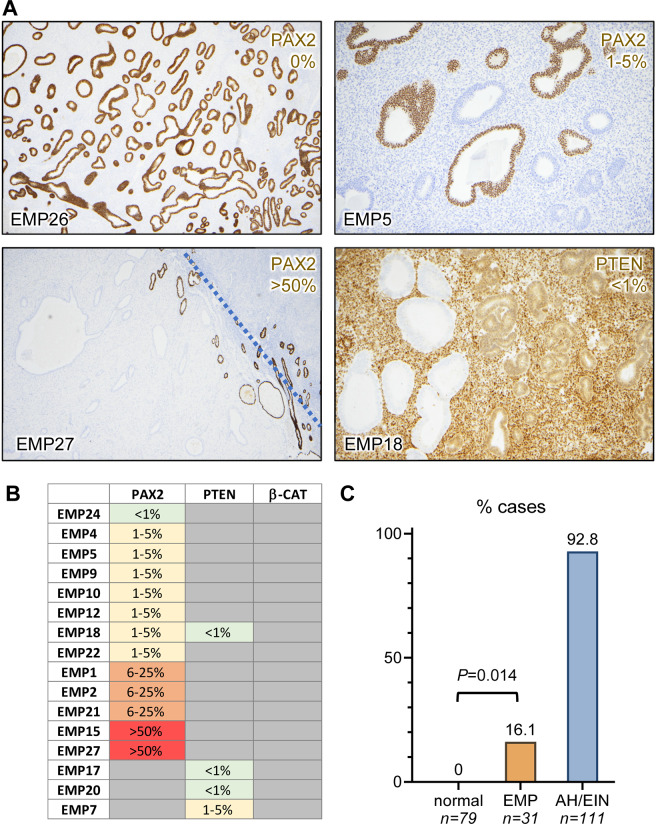
Analysis of EMPs with 3-marker panel. Tissue sections for all 31 EMPs were subjected to Pax2, Pten, and β-catenin IHC. **A** Representative images Pax2 and Pten, with percentage of overall loss in the sample in upper right-hand corners. For EMP27, the dashed blue line demarcates the boundary between the EMP and subjacent endomyometrium. Some glands retaining Pax2 are evident at the base of the EMP and in the subjacent endometrium. **B** Chart showing percent of aberrant glands for each of the three markers per previously established criteria^51^. **C** Bar graphs for aberrancy of any of the three markers in normal endometrium (*n* = 79), EMP (*n* = 31), and AH/EIN (*n* = 111). *P* value per Fisher’s exact test. Data for the normal endometria and AH/EIN were previously obtained^32^.

Recently, a 3-marker immunohistochemical (IHC) panel comprised of Pax2, Pten, and β-catenin was found to identify the great majority (93%) of endometrial precancers (AH/EIN)^32,41^. For Pax2 and Pten, loss of expression is a marker of AH/EIN, whereas for β-catenin, overexpression and nuclear localization is associated with premalignancy^42,43^. Thus, the 3-marker panel can serve as a surrogate to detect endometrial lesions likely to represent bona fide neoplasms. One nuance with the use of this panel is that Pax2- and Pten-deficient glands are present in some normal endometria, albeit in only a small proportion of the overall endometrium (usually <1%, but occasionally 1–5% of the total glands). Thus, we considered loss >5% to be a marker of endometrial neoplasia/premalignancy, as previously reported^32^. IHC for the 3 markers was performed on the 31 EMPs on the same tissue block used for DNA/RNA preparation. No EMPs showed definitive aberrance for β-catenin (Fig. 5B). Some cases showed small clones/regions of glandular Pten deficiency (Fig. 5A), but these were <1%, below the 5% cutoff that is a reliable indicator of premalignancy (Fig. 5B). In contrast, 5/31 (16.1%) of EMPs showed significant areas of Pax2 loss (*P* = 0.0014, Fisher’s exact test, 0/79 normal endometrial vs. 5/31 EMP) (Fig. 5A–C). Pax2 loss in the 5 EMPs (1, 2, 21, 15, 17 in Figs. 4C, 5B) did not correlate with gland density. Thus in summary, EMPs show a significant increase in the prevalence of Pax2 loss intermediate between normal endometria and definitive AH/EIN.

### Laser capture microdissection and digital PCR confirm epithelial origin of somatically-acquired mutations

**Fig. 6:**
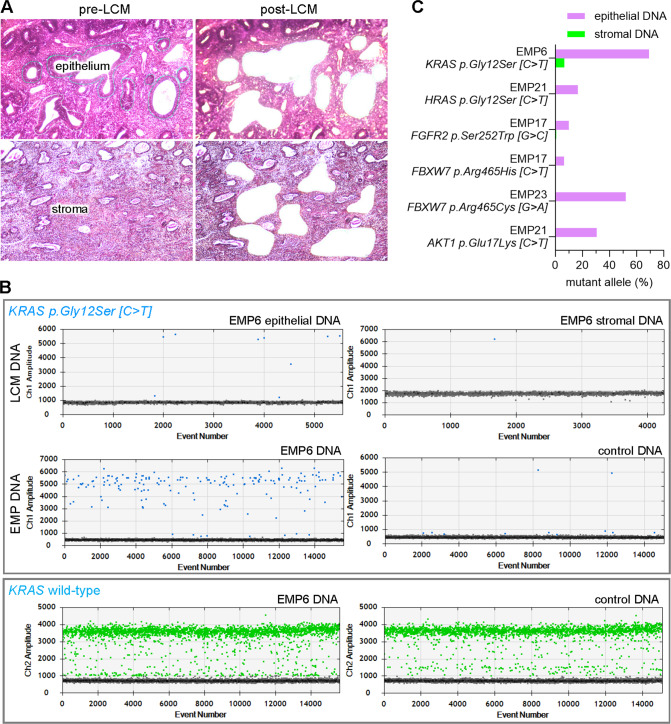
Laser capture microdissection (LCM)/digital PCR (dPCR) analysis of mutations in EMP epithelial versus stromal components. **A** Representative images of epithelial and stromal LCM. **B** Top panel shows representative dPCR analysis of mutant (FAM) probe specific for *KRAS p.Gly12Ser [C* > *T]* mutation. Middle panels are of non-LCM EMP6 and control EMP DNA samples (i.e., from non-microdissected tissue sections used for NGS). Bottom panel shows corresponding analysis with the wild-type *KRAS* allele (HEX) probe. **C** Bar graphs of mutant allele percentages of different cancer driver gene mutations in epithelial vs. stromal LCM samples.

Although the spectrum of observed mutations and its close resemblance to endometrial adenocarcinoma mutational spectra were consistent with epithelial origin, it remained a formal possibility that the mutations arose within the stromal compartment. To investigate this question, laser capture microdissection (LCM) was used to isolate stromal and epithelial compartments across tissue sections from six representative EMPs harboring mutations readily assayable by digital PCR (dPCR). LCM permitted precise dissection of glands and stroma from individual EMPs, with minimal cross-contamination (Fig. 6A). dPCR was performed on (1) the original DNA sample used for clinical NGS (non-microdissected positive control), (2) LCM epithelium, (3) LCM stroma, and (4) an NGS DNA sample from another EMP not harboring the mutation being assayed (negative control). Results are shown in detail for *KRAS p.Gly12Ser [C* > *T]* found in EMP6 (Fig. 6B). As expected, many more positive signals were detected in EMP6 than the negative control EMP (175 vs. 11, Fig. 6B, bottom row of top panel) whereas the wild-type probe yielded many more signals in EMP6 and control (Fig. 6B, bottom panel). Analysis of the LCM samples for the six EMPs consistently yielded more signals in the epithelial vs. stromal samples. The signal output from epithelial and stromal DNA was used to calculate the percent mutant allele and for the six mutations this was higher in the epithelial cells of the polyp, consistent with epithelial origin (Fig. 6C).

## Discussion

This study did not find evidence to support the presence of recurring chromosomal translocation(s) of presumptive mesenchymal/stromal origin in benign EMPs, which were reported in prior studies published in the 1990s^17–23^. In two of these studies, specific genes were implicated as the target of the rearrangements: *HMGIY*^19^ and *HMGI-C*^22^ (currently named *HMGA1* and *HMGA2* respectively per the HUGO Gene Nomenclature Committee^44^). Both *HMGA1* and *HMGA2* are represented in the gene panel used for this study. Of note, our NGS panel has routinely identified *HMGA2* translocations with diverse downstream partners e.g., *HMGA2::PTPRD*, *HMGA2::WIF1*, and *HMGA2::ACTR6* in three recent salivary gland tumors (carcinoma ex pleomorphic adenoma) that characteristically harbor *HMGA2* rearrangements. Thus, it seems unlikely that our assay would fail to detect *HMGA1/2* translocations. Furthermore, break-apart FISH conducted separately for both *HMGA1* and *HMGA2* failed to show any rearrangements in *n* = 16 EMPs. These results show that *HMGA1* and *HMGA2* are not defining or even common features of benign EMPs. However, we cannot exclude the possibility that chromosomal rearrangements involving unknown partners occur in sporadic EMPs. With this caveat, we found no evidence to support the notion that EMPs are mesenchymal outgrowths driven by gene fusions.

Tamoxifen administered for breast cancer is a risk factor both for EMPs and endometrial cancers. Three of the EMPs analyzed in this study were associated with a history of tamoxifen treatment (EMP 2, 4, 24) for ductal carcinoma-in-situ or invasive ductal carcinoma of the breast (Table S2). These 3 EMPs were of larger average size (6.2 cm) as compared to 2.8 cm for the remaining EMPs and were among the seven EMPs with the lowest gland:polyp ratios. These results are consistent with prior descriptions that tamoxifen-associated EMPs are larger and stroma-rich with relatively minor glandular components^5,45^. The 3 tamoxifen-associated EMPs were not distinctive with respect to their spectrum of definitive endometrial cancer driver mutations, which included *FGFR2*, *PIK3CA*, and *FBXW7* (Table S2). However, the documentation of endometrial cancer driver mutations in tamoxifen-associated EMPs together with the increased risk of endometrial cancer within EMPs^46^ further support the hysteroscopic resection of EMPs in this patient population. On a related note, the recurrence of EMPs is well-documented in some patients (<7%), sometimes related to tamoxifen use^47^. While many of the cases in this study had a diagnosis of EMP fragments on an endometrial biopsy, no patients had undergone a subsequent hysteroscopic polypectomy that could have established definitive EMP recurrence.

Unexpectedly, we identified a large number of point mutations in known oncogenic driver loci. While their low allelic frequencies might cast some doubt on their biological significance, several other observations suggest otherwise. First, there was a striking preponderance of known endometrial cancer genes among these loci, and unbiased analysis of the spectrum of these loci versus large cancer data sets identified endometrial cancers as the cancers with the most similar mutational spectrum. Second, many of these mutations produce amino acid substitutions that represent canonical, recurring, and well-understood oncogenic driver events. Third, dPCR of LCM epithelial and stromal samples confirmed that the mutations were of relatively low VAFs <5% and occurred within the epithelial compartment as would be expected for endometrial cancers, which are of epithelial origin^48,49^. Fourth, in a recent published study where we performed NGS on *n* = 19 normal endometrial controls, no endometrial cancer driver mutations were identified. A small number of variants of unknown biological significance were detected that likely represented age-related mutations, along with *MED12* leiomyomata driver mutations accounted for by the submucous leiomyomata in those cases^50,51^. The results of the two studies are not directly comparable because of different study designs, but the prior study was performed with a more limited endometrial-cancer specific panel with greater sensitivity to detect mutations at lower VAFs^51^.

Our findings lead us to propose a model where age-related mutations and in particular cancer-causing mutations accumulate in EMPs. In contrast to normal non-polypoid endometrium, which undergoes cyclical menstrual breakdown and shedding, we propose that EMPs, which are not shed, provide a “safe harbor” for these mutations. In this model, the mutations in endometrial cancer drivers are not the instigators driving the formation of the EMP, but rather accumulate as a function of age. This model explains the strong association between EMPs and cancers of both endometrioid and serous subtypes. Accordingly, our study identified mutations in genes that characterize endometrioid (e.g., *KRAS*, *PTEN*) or serous (*FBXW7*, *TP53*) cancers that commonly arise in EMPs^49,52–54^. Our results also provide further justification for hysteroscopic removal of endometrial polyps, when clinically feasible.

The above model is consistent with recent characterizations of the mutational landscape in normal human endometrial epithelium as a function of aging, which showed a bias towards oncogenic driver mutations including tumor suppressors and oncogenes with frequent mutations in *BRAF*, *HRAS*, *KRAS*, *PIK3CA*, *PIK3R1*, and *PTEN*, among others^55,56^ Consistent with these findings and our interpretations, age is the clinical and demographic factor most strongly associated with malignancy in EMPs including hysteroscopically resected polyps^15,46^. In one study of age-related mutations in normal (non-neoplastic) endometrium, analyses were performed on single microdissected glands. In most instances, mutations identified in a single microdissected gland were confined to that gland and not present in nearby glands^55^. Thus, it is not clear to what extent such mutations are eliminated by menstrual shedding, but it seems likely that their long-term persistence would require either colonization of basal glands that would represent a safe harbor in the context of shedding, since following menstruation, it is believed that only the basalis persists^57^. Also, while some age-related mutations do arise in premenopausal women^55^, their persistence is likely to be favored following the menopause.

The unique and abnormal microenvironment of the EMP might also favor tumor progression by other mechanisms in addition to the absence of menstrual shedding, which likely eliminates many age-related mutations^58^. For example, studies in postmenopausal women have revealed higher Ki67 mitotic indices in the epithelium of EMPs relative to adjacent endometrium^59–61^. Increased proliferation rate may be associated with telomere shortening, which can trigger rampant chromosomal instability and has been detected early in serous carcinogenesis in the fallopian tube and endometrium^62–64^. This may be a particularly relevant mechanism underlying serous carcinomas in EMPs. The reasons for increased proliferation in EMPs are not understood but may relate to a number of factors such as differences in effective hormone levels within the EMPs, perhaps as a consequence of their abnormal vascularization or stroma.

Another interesting finding in this study was the higher incidence of Pax2 loss in EMPs relative to normal endometria. Pax2 has received considerable attention as a practicable marker in the diagnosis of endometrial precancers (AH/EIN)^26,43,65–69^. Normal endometria can exhibit rare Pax2 loss, but significant areas of Pax2 loss (>5%) among endometrial glands is strongly correlated with AH/EIN. Pax2 is one component of a 3-marker panel (along with Pten and β-catenin) that can identify >90% of AH/EIN. We did not identify clear-cut Pten or β-catenin-aberrant EMPs, although Pten loss has been previously described in some EMPs^70^. Unlike Pten and β-catenin, which are frequently mutated in endometrial cancers and precancers, *PAX2* mutations have not been reported in endometrial cancers^43^. Whether Pax2 protein loss in EMPs signifies a loss of clonality or is a harbinger of malignancy requires further examination, but our results here suggest that in the clinical workup of AH/EIN in EMPs, Pax2 loss should be interpreted with caution as it occurs fairly often and in some cases over much larger areas than in normal endometria^32^.

Polypoid or exophytic lesions of the uterus that grossly mimic EMPs can represent bona fide mesenchymal neoplasms. Adenosarcomas are well-described malignancies that usually present as polypoid lesions of the cervix or endometrium^71,72^. By definition, such lesions are comprised of admixed malignant (sarcomatous) mesenchymal and benign epithelial components. Molecularly, adenosarcomas are characterized by loss-of-function mutations in *TP53* and *DICER1*, with frequent copy number variations in loci including *CDK4* and *MDM2*, among others^73–75^. A recent large next-generation sequencing study of atypical uterine polyps (polyps with atypical stromal features exhibiting some morphologic overlap with adenosarcomas) also confirmed focal gene amplifications of *CDK4* and *MDM2*, among other loci. We did not identify *CDK4* or *MDM2* gene amplifications, pointing to significant differences between EMPs and atypical uterine polyps/adenosarcomas. Interestingly, a small number of mutations in canonical endometrial cancer drivers (e.g., *KRAS*) were identified in this study of adenosarcomas. However, VAFs were not reported, making it unclear if these are significant driver events or “bystanders”, and whether they were stromal vs. epithelial was not explored. However, it seems likely that these oncogenic driver mutations were present in the epithelial component in agreement with our own results^76^. Another study specifically evaluating *RAS* mutations documented *KRAS* mutations in multiple EMPs. Although VAFs were not specifically reported, the results again echo our own findings^77^. Our study is unique, as far as we know, in its systematic analysis of all cancer genes (endometrial or otherwise) in entirely benign EMPs lacking atypical stromal or epithelial features histologically concerning for malignancy.

In summary, our study did not find evidence that EMPs are clonal neoplasms, but did provide compelling evidence that the strong association between EMPs and cancer is due to the accumulation of endometrial cancer driver mutations within EMPs, which bypass normal mechanisms of menstrual shedding.

## Supplementary information

Supplementary Figures 1 and 2

Supplementary Table S1

Supplementary Table S2
