# Supplementary material for: Endometrial polyps are non-neoplastic but harbor epithelial mutations in endometrial cancer drivers at low allelic frequencies
Source: Mod Pathol. 2022 Jul 7;35(11):1702–12. doi: 10.1038/s41379-022-01124-5 (PMC9596374; doi:10.1038/s41379-022-01124-5)
Supplement: Supplementary file 1 — Supplementary Figures 1 and 2 [file 41379_2022_1124_MOESM1_ESM.pdf]

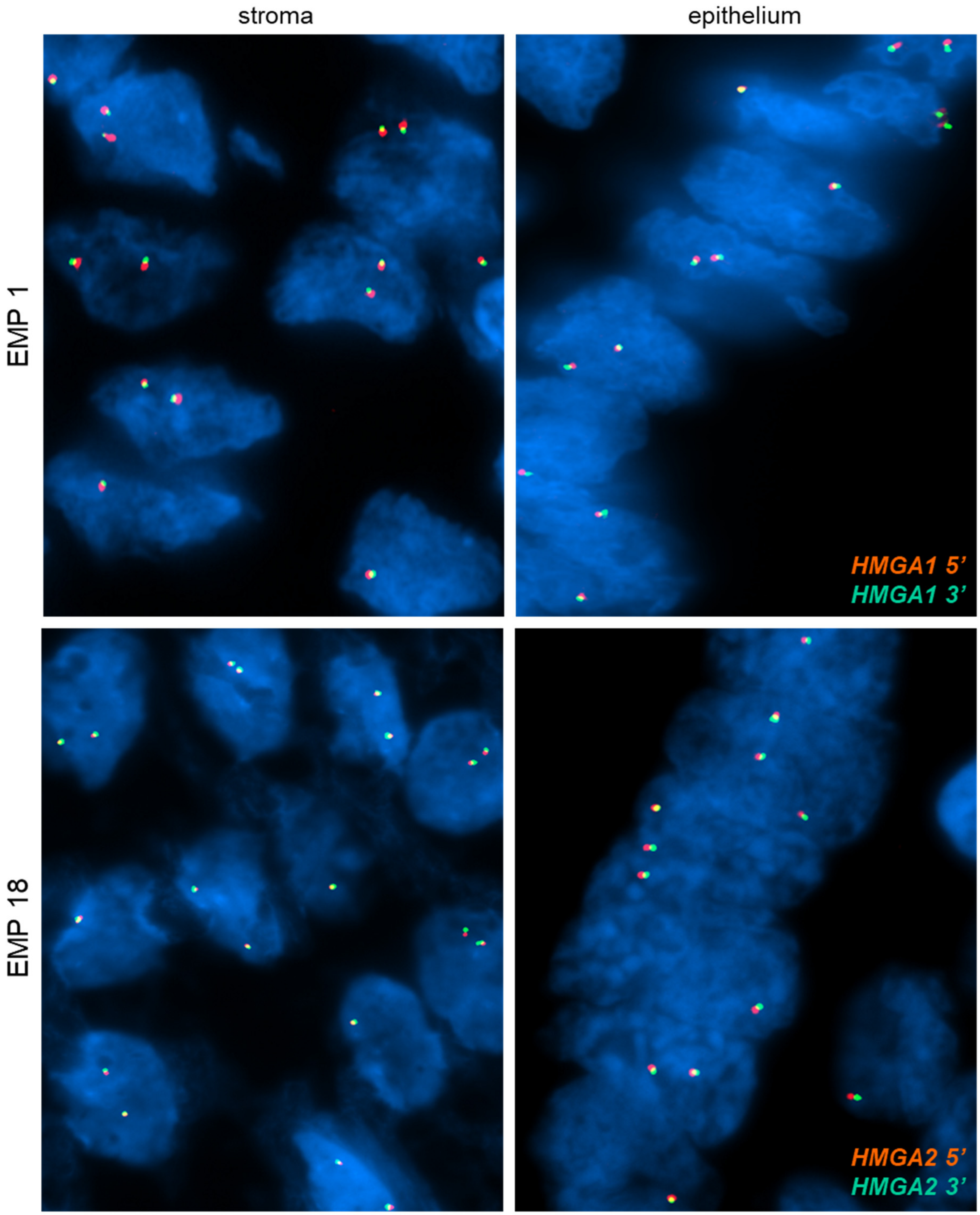

Supplementary Figure 1. Break-apart FISH for *HMGA1* and *HMGA2*. Hybdridization was performed on two separate sections (one per gene probe set) from 16 EMPs selected at random. Representative images are shown for two of the EMPs (1 and 18) for both the stroma and epithelium. The EMPs that were subjected to FISH were: 1, 3, 6, 12, 13, 14, 17, 18, 19, 22, 23, 25, 28, 29, 30, and 32. There were no rearrangements detected for either locus in either compartment in any of the 16 EMPs.

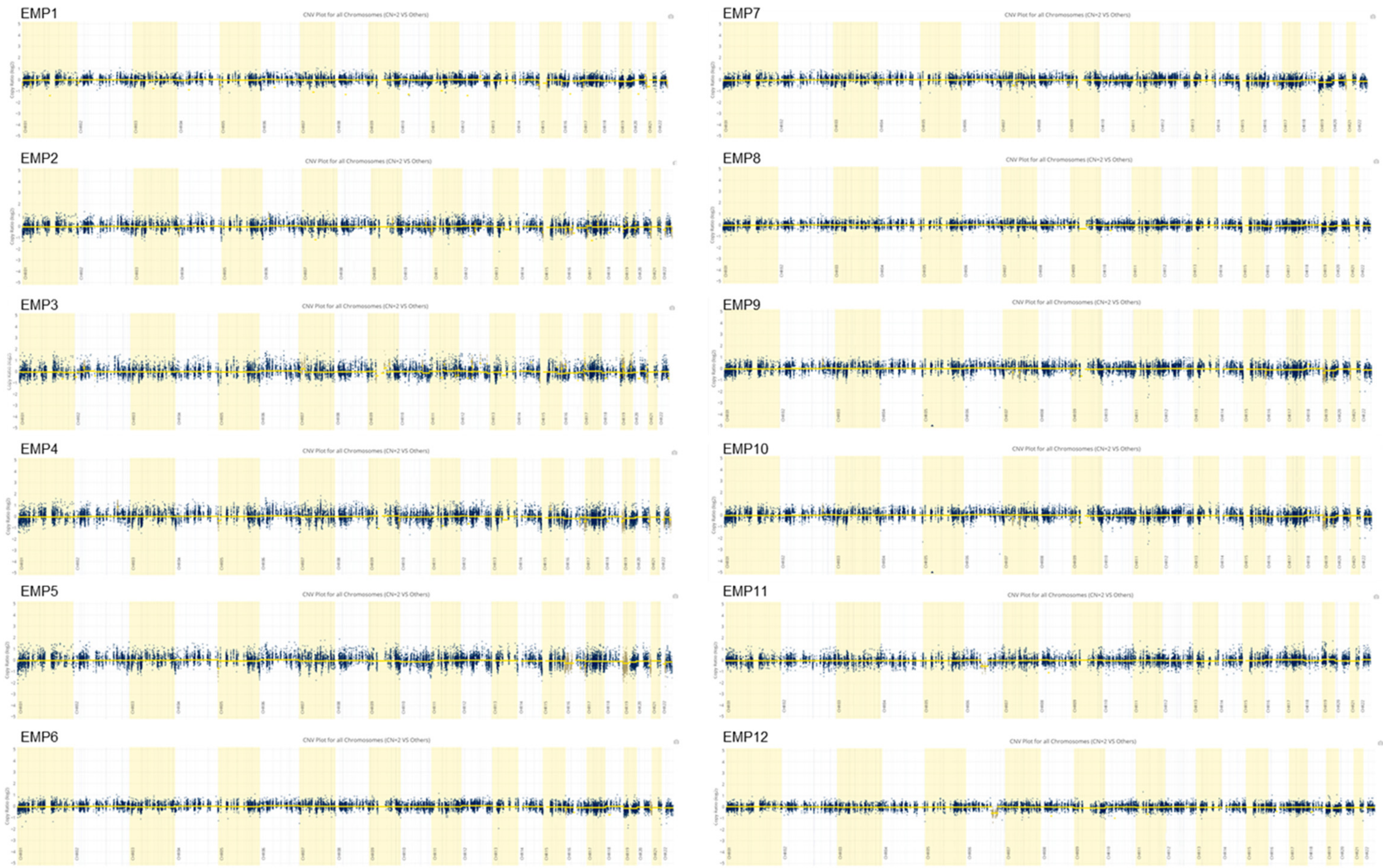

Supplementary Figure 2. Copy number plots based on DNA reads from next generation sequencing. For each sample the y-axis represents read depth (normalized to a panel of non-neoplastic normal samples). The x-axis represents position among the 22 autosomes as illustrated. Representative plots are shown (EMP1-12).
