# Supplementary material for: Endometrial polyps are non-neoplastic but harbor epithelial mutations in endometrial cancer drivers at low allelic frequencies
Source: Mod Pathol. 2022 Jul 7;35(11):1702–12. doi: 10.1038/s41379-022-01124-5 (PMC9596374; doi:10.1038/s41379-022-01124-5)
Supplement: Supplementary file 2 — Supplementary Table S1 [file 41379_2022_1124_MOESM2_ESM.pdf]

UT SOUTHWESTERN NEXT GENERATION SEQUENCING PAN-CANCER MUTATION TEST GENE LIST (n=1516 loci)

ABCC3	ANKRD26	BAIAP2L1	C11orf1	CCT6B	CENPU	CREB3L2	DDX6	EGR2	ETV5	FGF3
ABI1	ANKRD28	BAP1	C11orf30	CD19	CEP170B	CREBBP	DEK	EGR3	ETV6	FGF4
ABL1	ANLN	BARD1	C11orf54	CD22	CEP57	CRKL	DGKB	EGR4	EWSR1	FGF5
ABL2	APC	BAX	C11orf95	CD274	CEP85L	CRLF2	DGKI	EIF4A2	EXO1	FGF6
ABLIM1	APH1A	BAZ2A	C2CD2L	CD28	CHCHD7	CRTC1	DGKZ	EIF4E	EXOSC6	FGF7
ABRAXAS1	APLP2	BCAS3	C2orf44	CD36	CHD2	CRTC3	DICER1	ELF4	EXT1	FGF8
ACACA	APOD	BCAS4	CACNA1F	CD44	CHD6	CSF1	DIRAS3	ELK4	EXT2	FGF9
ACE	AR	BCL10	CACNA1G	CD58	CHEK1	CSF1R	DIS3L2	ELL	EYA1	FGFR1
ACER1	ARAF	BCL11A	CACNA2D3	CD70	CHEK2	CSF3	DKK1	ELN	EYA2	FGFR1OP
ACKR3	ARFRP1	BCL11B	CAD	CD74	CHIC2	CSF3R	DKK2	ELOVL2	EZH2	FGFR1OP2
ACSBG1	ARHGAP20	BCL2	CALR	CD79A	CHL1	CSNK1G2	DKK4	ELP2	EZR	FGFR2
ACSL3	ARHGAP26	BCL2A1	CAMK2A	CD79B	CHMP2B	CSNK2A1	DLEC1	EML1	FAF1	FGFR3
ACSL6	ARHGEF12	BCL2L1	CAMK2B	CD8A	CHN1	CTCF	DLL1	EML4	FAM127C	FGFR4
ACVR1B	ARHGEF7	BCL2L2	CAMK2G	CDC14A	CHST11	CTDSP2	DLL3	ENPP2	FAM19A2	FH
ACVR1C	ARID1A	BCL3	CAMTA1	CDC14B	CHUK	CTLA4	DLL4	EP300	FAM19A5	FHIT
ACVR2A	ARID2	BCL6	CANT1	CDC25A	CIC	CTNNA1	DMRT1	EP400	FAM46C	FHL2
ADD3	ARIH2	BCL7A	CAPRIN1	CDC25C	CIITA	CTNNB1	DMRTA2	EPC1	FAM64A	FIGF
ADGRA2	ARL6IP5	BCL9	CAPZB	CDC42	CIRH1A	CTNND2	DNAJB1	EPCAM	FANCA	FIP1L1
ADGRG7	ARNT	BCOR	CARD11	CDC73	CIT	CTRB1	DNM1	EPHA10	FANCB	FLCN
ADM	ARRDC4	BCORL1	CARM1	CDH1	CKB	CTSA	DNM2	EPHA2	FANCC	FLI1
AFF1	ASMTL	BCR	CARS	CDH11	CKS1B	CUL4A	DNM3	EPHA3	FANCD2	FLNA
AFF3	ASPH	BDNF	CASC5	CDK1	CLP1	CUL4B	DNMT1	EPHA5	FANCE	FLNC
AFF4	ASPSCR1	BHLHE22	CASP3	CDK12	CLTA	CUX1	DNMT3A	EPHA7	FANCF	FLT1
AGR3	ASTN2	BICC1	CASP7	CDK2	CLTC	CXCL8	DOCK1	EPHB1	FANCG	FLT3
AHCYL1	ASXL1	BIN1	CASP8	CDK4	CLTCL1	CXCR4	DOT1L	EPHB6	FANCI	FLT3LG
AHI1	ATF1	BIRC3	CAV1	CDK5RAP2	CMKLR1	CXXC4	DPEP3	EPO	FANCL	FLT4
AHR	ATF3	BIRC6	CBFA2T3	CDK6	CNBP	CYFIP2	DPM1	EPOR	FANCM	FLYWCH1
AHRR	ATG13	BLM	CBFB	CDK7	CNOT2	CYLD	DPYD	EPS15	FAS	FNBP1
AIP	ATG5	BMP4	CBL	CDK8	CNTN1	CYP1B1	DST	ERBB2	FASLG	FOS
AK2	ATIC	BMPR1A	CBLB	CDK9	CNTRL	CYP2C19	DTX1	ERBB3	FBN2	FOSB
AK5	ATL1	BRAF	CBLC	CDKL5	COG5	DAB2IP	DTX4	ERBB4	FBXO11	FOSL1
AKAP12	ATM	BRCA1	CCAR2	CDKN1A	COL11A1	DACH1	DUSP2	ERC1	FBXO31	FOXL2
AKAP6	ATP1B4	BRCA2	CCDC28A	CDKN1B	COL1A1	DACH2	DUSP22	ERCC1	FBXW7	FOXO1
AKAP9	ATP8A2	BRD1	CCDC6	CDKN1C	COL1A2	DAXX	DUSP26	ERCC2	FCGBP	FOXO3
AKR1C3	ATR	BRD3	CCDC88C	CDKN2A	COL3A1	DCLK2	DUSP9	ERCC3	FCGR2B	FOXO4
AKT1	ATRNL1	BRD4	CCK	CDKN2B	COL6A3	DCN	E2F1	ERCC4	FCRL4	FOXP1
AKT2	ATRX	BRIP1	CCL2	CDKN2C	COL9A3	DDB2	EBF1	ERCC5	FEN1	FRK
AKT3	AURKA	BRSK1	CCNA2	CDKN2D	COMMD1	DDIT3	ECT2L	ERCC6	FEV	FRMPD4
ALDH1A1	AURKB	BRWD3	CCNB1IP1	CDX1	COX6C	DDR2	EDIL3	ERG	FGF1	FRS2
ALDH2	AUTS2	BTBD18	CCNB3	CDX2	CPNE1	DDX10	EDNRB	ERLIN2	FGF10	FRYL
ALDOC	AXIN1	BTG1	CCND1	CEBPA	CPS1	DDX20	EED	ESR1	FGF13	FSTL3
ALK	AXL	BTG2	CCND2	CEBPB	CPSF6	DDX39B	EEFSEC	ETS1	FGF14	FUS
AMER1	BACH1	BTK	CCND3	CEBPD	CRADD	DDX3X	EGF	ETS2	FGF19	FUT1
AMH	BACH2	BTLA	CCNE1	CEBPE	CREB1	DDX41	EGFR	ETV1	FGF2	FZD10
ANGPT1	BAG4	BUB1B	CCNG1	CENPF	CREB3L1	DDX5	EGR1	ETV4	FGF23	FZD2

FZD3	GRIN2B	HMGB1	IL13RA2	KDM2B	LINGO2	MAP3K7	MLLT4	NDC80	NUP214	PDGFRB
FZD6	GRM1	HMGN2P46	IL15	KDM4C	LMBRD1	MAPK1	MLLT6	NDE1	NUP93	PDK1
FZD7	GRM3	HNF1A	IL1B	KDM5A	LMO1	MAPK3	MMP7	NDRG1	NUP98	PEG3
FZD8	GSK3B	HNRNPA2B1	IL1R1	KDM5C	LMO2	MAPK8	MMP9	NDUFAF1	NUTM1	PER1
GAB1	GSN	HOOK3	IL1RAP	KDM6A	LMO7	MAPK8IP2	MN1	NEDD4	NUTM2A	PFDN5
GABRG2	GTF2I	HOXA10	IL2	KDR	LNP1	MAPK9	MNAT1	NEURL1	NUTM2B	PHB
GADD45B	GTSE1	HOXA11	IL21R	KDSR	LOX	MAPRE1	MNX1	NF1	OFD1	PHF1
GANAB	H2AFX	HOXA13	IL2RA	KEAP1	LPAR1	MATK	MPL	NF2	OLIG1	PHF23
GAS1	H3F3A	HOXA3	IL3	KIAA0232	LPP	MAX	MRE11A	NFATC1	OLIG2	PHF6
GAS5	HAS2	HOXA9	IL6	KIAA1524	LPXN	MB21D2	MSH2	NFATC2	OLR1	PHOX2B
GAS7	HDAC1	HOXC11	IL7R	KIAA1549	LRIG3	MBNL1	MSH3	NFE2L2	OMD	PI4KA
GATA1	HDAC2	HOXC13	INHBA	KIAA1598	LRMP	MBTD1	MSH6	NFIB	P2RY8	PICALM
GATA2	HDAC3	HOXD11	INPP4A	KIF5B	LRP1B	MCL1	MSI2	NFKB1	PAFAH1B2	PIK3CA
GATA3	HDAC4	HOXD13	INPP4B	KIT	LRP5	MDC1	MSN	NFKB2	PAG1	PIK3CB
GATA6	HDAC5	HOXD9	INPP5A	KLF4	LRPPRC	MDH1	MST1R	NFKBIA	PAK1	PIK3CD
GBP2	HDAC6	HRAS	INPP5D	KLHL6	LRRC37B	MDM2	MTCP1	NGF	PAK3	PIK3CG
GDF6	HDAC7	HSP90AA1	IQCG	KLK2	LRRC59	MDM4	MTOR	NGFR	PAK6	PIK3R1
GEN1	HECW1	HSP90AB1	IRF1	KLK7	LRRC7	MDS2	MTUS2	NIN	PAK7	PIK3R2
GFAP	HEPH	HSPA1A	IRF2BP2	KMT2A	LRRK2	MEAF6	MUC1	NIPBL	PALB2	PIM1
GHR	HERPUD1	HSPA2	IRF4	KMT2B	LTBP1	MECOM	MUTYH	NKX2-1	PAPPA	PKM
GID4	HES1	HSPA4	IRF8	KMT2C	LYL1	MED12	MYB	NKX2-5	PARP2	PLA2G2A
GIT2	HES5	HSPA5	IRS1	KMT2D	LYN	MEF2B	MYBL1	NOD1	PARP3	PLA2G5
GLI1	HEY1	HTRA1	IRS2	KNSTRN	MACROD1	MEF2C	MYC	NODAL	PARP4	PLAG1
GLI2	HGF	HUWE1	IRS4	KPNB1	MAD2L1	MEF2D	MYCL	NONO	PASK	PLAT
GLI3	HHEX	IBSP	ITGA5	KRAS	MADD	MELK	MYCN	NOS3	PATZ1	PLAU
GMPS	HIF1A	ICAM1	ITGA7	KSR1	MAF	MEN1	MYD88	NOTCH1	PAX3	PLCB1
GNA11	HIP1	ICK	ITGA8	KTN1	MAFB	MET	MYH11	NOTCH2	PAX5	PLCB4
GNA12	HIPK1	ID1	ITGAV	LAMA1	MAGED1	METTL18	MYH9	NOTCH3	PAX7	PLCG1
GNA13	HIPK2	ID3	ITGB3	LAMA5	MAGEE1	METTL7B	MYO18A	NOTCH4	PAX8	PLCG2
GNAI1	HIST1H1C	ID4	ITK	LAMP2	MAGOH	MFNG	MYO1F	NPM1	PBRM1	PLEKHM2
GNAQ	HIST1H1D	IDH1	ITPKA	LASP1	MALAT1	MGEA5	NAB2	NPM2	PBX1	PML
GNAS	HIST1H1E	IDH2	JAG2	LCK	MALT1	MGMT	NACA	NR3C1	PC	PMS1
GNG4	HIST1H2AC	IFNG	JAK1	LCP1	MAML1	MIB1	NAPA	NR4A3	PCBP1	PMS2
GOLGA5	HIST1H2AG	IFRD1	JAK2	LEF1	MAML2	MIPOL1	NAV3	NR6A1	PCLO	POFUT1
GOPC	HIST1H2AL	IGF1	JAK3	LEFTY2	MAP2	MITF	NBEAP1	NRAS	PCM1	POLD1
GOSR1	HIST1H2AM	IGF1R	JARID2	LFNG	MAP2K1	MKI67	NBN	NRG1	PCNA	POLD2
GOT1	HIST1H2BC	IGFBP2	JAZF1	LGALS3	MAP2K2	MKL1	NBR1	NSD1	PCSK7	POLD3
GPC3	HIST1H2BJ	IGFBP3	JUN	LGR5	MAP2K3	MKL2	NCAM1	NT5C2	PDCD1	POLD4
GPHN	HIST1H2BK	IKBKB	KALRN	LHFP	MAP2K4	MLF1	NCKIPSD	NTF3	PDCD11	POLE
GPR34	HIST1H2BO	IKBKE	KANK1	LHX2	MAP2K5	MLH1	NCOA1	NTF4	PDCD1LG2	POLQ
GRB10	HIST1H3B	IKZF1	KAT2B	LHX4	MAP2K6	MLH3	NCOA2	NTRK1	PDE4DIP	POLR2H
GRB2	HIST1H4I	IKZF2	KAT6A	LIFR	MAP2K7	MLLT1	NCOA3	NTRK2	PDGFA	POM121
GRHPR	HLF	IKZF3	KAT6B	LINC00598	MAP3K1	MLLT10	NCOA4	NTRK3	PDGFB	POMGNT1
GRID1	HMGA1	IL12RB2	KCNB1	LINC00982	MAP3K14	MLLT11	NCOR2	NUMA1	PDGFD	POSTN
GRIN2A	HMGA2	IL13	KDM1A	LINC01565	MAP3K6	MLLT3	NCSTN	NUP107	PDGFRA	POT1

POU2AF1	PRSS8	RB1	RRM1	SIK3	SPRY4	TAL2	TMEM30A	UFM1	XRCC2
POU5F1	PSD3	RBM15	RRM2B	SIN3A	SPTAN1	TAOK1	TMPRSS2	USP16	XRCC3
PPAP2B	PSEN1	RBM6	RTEL1	SIRT1	SPTBN1	TBL1XR1	TNC	USP42	XRCC6
PPARG	PSIP1	RCHY1	RTN3	SKP2	SQSTM1	TBX15	TNF	USP5	YAP1
PPARGC1A	PSMD2	RCOR1	RUNX1	SLC1A2	SRC	TCEA1	TNFAIP3	USP6	YPEL5
PPFIA2	PTBP1	RCSD1	RUNX1T1	SLC34A2	SRF	TCF12	TNFRSF10B	USP7	YTHDF2
PPFIBP1	PTCH1	RECQL4	RUNX2	SLC45A3	SRGAP3	TCF3	TNFRSF10D	VCAM1	YWHAE
PPM1D	PTCRA	REEP3	RYR3	SLC7A5	SRP72	TCF7L2	TNFRSF11A	VEGFA	YY1AP1
PPP1CB	PTEN	RELA	S1PR2	SLCO1B3	SRRM3	TCL1A	TNFRSF14	VEGFC	ZBTB16
PPP1R13B	PTGS2	RELN	SAMD9	SLX4	SRSF2	TCL6	TNFRSF17	VGLL3	ZC3H7A
PPP1R13L	PTK2	RERG	SAMD9L	SMAD2	SRSF3	TCTA	TNFRSF6B	VHL	ZC3H7B
PPP2CB	PTK2B	RET	SARNP	SMAD3	SS18	TEAD1	TOP1	VTI1A	ZFP64
PPP2R1A	PTK7	RFC1	SBDS	SMAD4	SS18L1	TEAD2	TOP2A	WASF2	ZFPM2
PPP2R1B	PTPN11	RFC2	SCN8A	SMAD6	SSBP1	TEAD3	TOP2B	WDFY3	ZFYVE19
PPP2R2A	PTPN2	RFC3	SDC4	SMAP1	SSBP2	TEAD4	TP53	WDR1	ZIC2
PPP2R2B	PTPN6	RFC4	SDHA	SMARCA1	SSX1	TEC	TP53BP1	WDR18	ZMIZ1
PPP2R4	PTPRA	RFC5	SDHAF2	SMARCA4	SSX2	TENM1	TP63	WDR70	ZMYM2
PPP3CA	PTPRK	RGS7	SDHB	SMARCA5	SSX4	TERF1	TP73	WDR90	ZMYM3
PPP3CB	PTPRO	RHBDF2	SDHC	SMARCB1	ST6GAL1	TERF2	TPD52L2	WEE1	ZMYND11
PPP3CC	PTPRR	RHEB	SDHD	SMC1A	STAG2	TERT	TPM3	WHSC1	ZNF207
PPP3R1	PTTG1	RHOA	SEC31A	SMC3	STAT1	TET1	TPM4	WHSC1L1	ZNF217
PPP3R2	PVT1	RHOD	SEPT2	SMO	STAT3	TET2	TPO	WIF1	ZNF24
PPP4C	RABEP1	RHOH	SEPT5	SNAPC3	STAT4	TFAP2A	TPR	WISP3	ZNF331
PQLC3	RAC1	RICTOR	SEPT6	SNCG	STAT5A	TFDP1	TRAF2	WNT10A	ZNF384
PRCC	RAC2	RLTPR	SEPT9	SNHG5	STAT5B	TFE3	TRAF3	WNT10B	ZNF444
PRDM1	RAC3	RMI2	SERP2	SNW1	STAT6	TFEB	TRAF5	WNT11	ZNF521
PRDM16	RAD21	RNF213	SERPINE1	SNX29	STIL	TFG	TRHDE	WNT16	ZNF585B
PRDM7	RAD50	RNF217-AS1	SERPINF1	SNX9	STK11	TFPT	TRIM24	WNT2B	ZNF687
PRF1	RAD51	RNF43	SET	SOCS1	STRN	TFRC	TRIM27	WNT3	ZNF703
PRG2	RAD51B	ROBO1	SETBP1	SOCS2	STX5	TGFB2	TRIM33	WNT4	ZRSR2
PRICKLE1	RAD51C	ROBO2	SETD2	SOCS3	STYK1	TGFB3	TRIP11	WNT5B	
PRKACA	RAD51D	ROS1	SETD7	SOD2	SUFU	TGFBI	TRPS1	WNT6	
PRKACG	RAD52	RP11-146B14.1	SF3B1	SORBS2	SUGP2	TGFBR2	TSC1	WNT7B	
PRKAR1A	RAD54L	RPA1	SFPQ	SORT1	SULF1	TGFBR3	TSC2	WNT8B	
PRKCA	RAF1	RPA3	SFRP2	SOS1	SUV39H2	THADA	TSHR	WRN	
PRKCB	RALGDS	RPL22	SFRP4	SOX10	SUZ12	THBS1	TTK	WSB1	
PRKCD	RANBP17	RPN1	SGK1	SOX11	SYK	THRAP3	TTL	WT1	
PRKCG	RANBP2	RPN2	SGPP2	SOX2	SYP	TIAM1	TUSC3	WWOX	
PRKDC	RAP1GDS1	RPS21	SH2D5	SP1	TACC1	TIRAP	TYK2	WWTR1	
PRKG2	RARA	RPS6KA1	SH3BP1	SP3	TACC2	TLL2	TYMS	XBP1	
PRMT1	RASAL1	RPS6KA2	SH3D19	SPECC1	TACC3	TLR4	U2AF1	XIAP	
PRMT8	RASGEF1A	RPS6KA3	SH3GL1	SPEN	TAF1	TLX1	U2AF2	XKR3	
PROM1	RASGRF1	RPS6KB1	SH3GL2	SPOP	TAF15	TLX3	UBE2B	XPA	
PRRX1	RASGRF2	RPTOR	SHC1	SPP1	TAF1L	TMEM127	UBE2C	XPC	
PRRX2	RASGRP1	RREB1	SHC2	SPRY2	TAL1	TMEM230	UFC1	XPO1	
